# Core-level spectra and molecular deformation in adsorption: V-shaped pentacene on Al(001)

**DOI:** 10.3762/bjnano.6.230

**Published:** 2015-11-27

**Authors:** Anu Baby, He Lin, Gian Paolo Brivio, Luca Floreano, Guido Fratesi

**Affiliations:** 1 ETSF, CNISM, Dipartimento di Scienza dei Materiali, Università di Milano-Bicocca, Via Cozzi 55, I-20125 Milano, Italy; 2 CNR-IOM, Laboratorio TASC, Basovizza SS-14, Km 163.5, I-34149 Trieste, Italy; 3 Dipartimento di Fisica, Università degli Studi di Milano, Via Celoria 16, I-20133 Milano, Italy

**Keywords:** aluminum, density functional theory (DFT), near-edge X-ray absorption fine structure (NEXAFS), pentacene, X-ray photoelectron spectroscopy (XPS)

## Abstract

By first-principle simulations we study the effects of molecular deformation on the electronic and spectroscopic properties as it occurs for pentacene adsorbed on the most stable site of Al(001). The rationale for the particular V-shaped deformed structure is discussed and understood. The molecule–surface bond is made evident by mapping the charge redistribution. Upon X-ray photoelectron spectroscopy (XPS) from the molecule, the bond with the surface is destabilized by the electron density rearrangement to screen the core hole. This destabilization depends on the ionized carbon atom, inducing a narrowing of the XPS spectrum with respect to the molecules adsorbed hypothetically undistorted, in full agreement to experiments. When looking instead at the near-edge X-ray absorption fine structure (NEXAFS) spectra, individual contributions from the non-equivalent C atoms provide evidence of the molecular orbital filling, hybridization, and interchange induced by distortion. The alteration of the C–C bond lengths due to the V-shaped bending decreases by a factor of two the azimuthal dichroism of NEXAFS spectra, i.e., the energy splitting of the sigma resonances measured along the two in-plane molecular axes.

## Introduction

Pentacene has been studied extensively as it is a potential candidate in the field of organic electronic devices [1–5]. It acts as a p-type organic semiconductor in its intrinsic state with high hole mobility and exhibits a very high melting point [6]. The pentacene–Al junction is known to exhibit a Schottky barrier and, hence, finds numerous applications in the manufacturing of diodes, transistors and other devices [7–12]. Despite of these interesting applications, only very few basic studies have been done on this system. In particular the challenges in the preparation of a well-ordered Al surface might have hindered the experimental investigations, while the previous ab initio theoretical studies [13–14] on this system were missing long-range van der Waals (vdW) corrections. Simeoni and Picozzi reported a numerical investigation of pentacene on Al(001) by density functional theory (DFT) with the local density approximation (LDA) and the generalized gradient approximation (GGA) [13]. They found that the interaction between pentacene and Al is rather weak and adsorption occurs at a height from the surface of about 5.71 Bohr (3.02 Å) in LDA and of 7.20 Bohr (3.81 Å) in GGA, with a molecular corrugation of 0.74 Å in LDA. The same system was later investigated with LDA and GGA by Saranya et al. [14] who also obtained a very weak adsorption energy with pentacene adsorbed at larger distances from the Al surface (3.4 Å) in LDA and comparable to the previous ones in GGA. Both papers also report Schottky barriers at the junction due to the interfacial electron transfer.

In our recent work [15], we studied experimentally and theoretically the adsorption of pentacene on the Al(001) substrate. We performed simulations including the long range vdW interactions and without them. In the latter case we observed that the bonding energy is clearly underestimated. In our calculations including vdW, the most stable adsorption site is found to be the bridge (B) site where the adsorbed pentacene is bent around the central C atoms, which are more strongly bound to the surface Al atoms, forming a V-shape with the long molecular axis aligned along the [110] direction [15]. A similar V-shaped deformation was also obtained in the configuration with the long molecular axis along the [010] direction but with 0.42 eV higher adsorption energy. On the contrary, other adsorption configurations would result in planar molecular geometries with higher adsorption energies (at least by 0.7 eV) and hence are considered unphysical. Scanning tunneling microscopy (STM) measurements showed that a large percentage of pentacene molecules adsorb with a V-shape on a reconstructed Al(001) surface with the longer axis along the [110] direction. The calculated results of XPS and NEXAFS assuming the V-shaped adsorption are in agreement with the experiments. For comparison only a minor bending of the molecule was reported in experimental and theoretical studies of pentacene on Au(111) [16], Cu(110) [17], and Cu(001) [18], while an asymmetric adsorption along the long edge was determined for Co islands on Cu(111) [19].

The peculiar V-shaped bending attained in our work [15] is a very interesting feature, which was never reported before and whose influence on the electronic and spectroscopic properties of the interface is investigated in this paper. We evaluate by DFT the screening charges of the adsorbed system and relate them to the deformation of pentacene. Comparison of the results with those obtained for the undistorted non-physical adsorption at top (T) site allows for a better understanding of the system properties. The contributions to XPS and NEXAFS of non-equivalent carbon atoms in which a 1s core hole is created are calculated. Together with the screening charges they allow for a detailed understanding of the spectral features as modified by the molecular V-shaped distortion.

## Results and Discussion

### Origin of the V-shape

**Figure 1:**
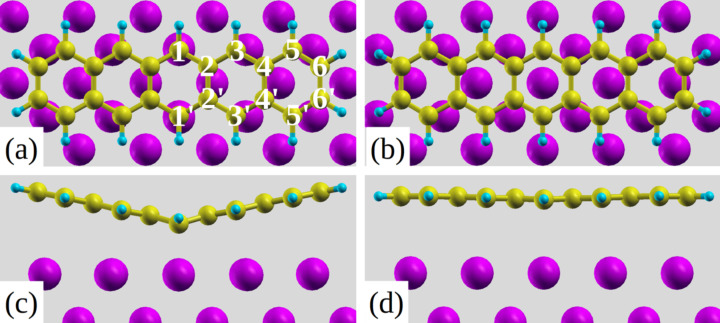
Top views (a), (b) and side views (c), (d) of bridge (B) and top (T) sites, respectively, where (a) also shows the numbering of the C atoms.

In Baby et al. we showed that the most stable bonding configuration of pentacene on Al(001) is the B-site [15]. As depicted in Figure 1a,c this configuration is highly distorted around the central C atoms (1,1’) so as to bind on top of the two surface Al atoms at the short C–Al distance of 2.20 Å. As a comparison, Figure 1b,d show the undistorted adsorption configuration as it is calculated at a T site. Bending at the central position is in agreement with the findings about the reactivity of pentacene and related molecules [20], as described in terms of the molecular aromaticity (defined as the ability of the π-electrons to delocalize above and below the plane of cyclic molecules resulting in their extra stabilization [21–22]). If we consider benzene the most aromatic molecule as an example, as more rings are added to it, the electron density within the molecule rearranges in such a manner that it is largest for the central ring (hence highly reactive even if more aromatic) and decreases towards the outer ones [23–24]. Taking part in reactions through the most aromatic central ring is convenient for the molecule as the activation energy barrier is lower [25]. Furthermore the HOMO coefficients are highest for the central carbon atoms in acenes and decrease towards the outer ring ones [25]. Other studies related reactivity to the ring current showing that this is highest for the central ring in the case of pentacene and also the highest proton chemical shift is found for the hydrogen atoms attached to the central carbon atoms in the case of acenes [26]. All these results support the reactive nature of the central carbon atoms in pentacene. In agreement with such properties we verified that V-shaped pentacene bonds to the Al atoms underneath through the two central carbon atoms of the innermost phenyl ring as the distance between them (C1–C1’, see Figure 1a for numbering) becomes comparable to the Al–Al one.

### Effects of V-shape on bond length

The V-shaped molecular deformation influences the carbon–carbon bond lengths of adsorbed pentacene. This is reported in Table 1, in which the bond lengths of the free molecule in the gas phase (g) are compared to those of the molecule adsorbed flat and bent at T and B sites, respectively. A common trend is found while moving from the free molecule to the flat and to the bent adsorbed molecule, as they keep either increasing or decreasing, conversely decreasing or increasing the π-character of those bonds [27]. Note that the bond lengths for g and T cases are very similar and different from those of the B configuration. Due to the strong coupling between C1 and the underlying Al atom at the B site the C1–C2 bond experiences the highest variation in length, which increases by 0.07 Å from g to B. Such an elongation occurs because the C1 orbital modifies from sp^2^ to sp^3^ hybridization, which results in a displacement of the valence electron density of C1 from the C1–C2 bond to the newly formed C1–Al bond, as recently discussed for self-assembled monolayers [28]. This further affects the delicate electron density balance within the molecule, i.e., while the C1–C2 bond length increases (decreasing π-character), the C2–C2’ bond length decreases (increasing π-character) at the B site. This kind of rearrangement in the electron density reduces the aromaticity of the central carbon ring of pentacene but in a smaller way than that for bending through the outer rings [25], as discussed before. Furthermore we observe shorter C2–C2’, C4–C4’ and C6–C6’ bond lengths (with an average of about 0.02 Å) moving from second to fourth column of Table 1 additionally indicating that the molecule shrinks along the short molecular axis. On the contrary an increase in the length of the molecule is observed along the long axis in the B configuration hinting a slight weakening in the coupling of outer atoms with respect to those of pentacene in gas phase. Summarizing, the differences between the bond lengths in the two directions reduces.

**Table 1:** Carbon–carbon bond lengths for free pentacene (g) and for pentacene adsorbed at the top (T) and the bridge (B) site.

C–C	g (Å)	T (Å)	B (Å)

C1–C2	1.400	1.408	1.466
C2–C3	1.411	1.409	1.396
C3–C4	1.390	1.400	1.420
C4–C5	1.429	1.423	1.414
C5–C6	1.368	1.375	1.383

average	1.400	1.403	1.416

C2–C2’	1.458	1.454	1.428
C4–C4’	1.453	1.448	1.428
C6–C6’	1.427	1.422	1.410

average	1.446	1.441	1.422

### Ground-state electronic properties and charge transfer

**Figure 2:**
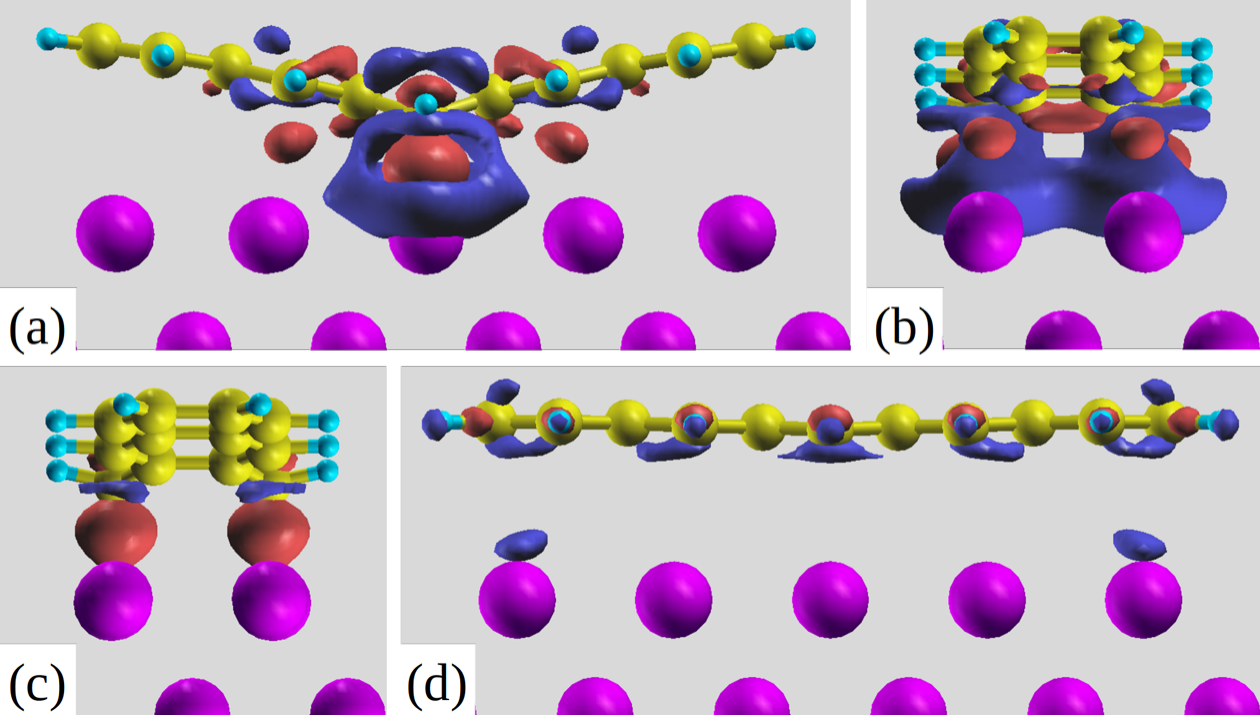
(a,b) Side views of the isodensity plot showing the bond charge of pentacene at the B-site for an isodensity = 0.002 *e*/Bohr^3^ (red regions: high electron density and blue regions: low electron density), (c) pentacene at the B-site for an isodensity of 0.005 *e*/Bohr^3^, (d) pentacene at the T-site for an isodensity of 0.001 *e*/Bohr^3^.

We now quantify the interactions taking place between the molecule and the metal substrate in terms of charge transfer upon adsorption. The isodensity plots showing the three-dimensional charge rearrangements for pentacene at B and T sites are shown in Figure 2, which can be defined using the equation Δρ = ρ(pentacene/Al) − ρ(pentacene) − ρ(Al), where ρ(pentacene/Al) is the total charge of the combined system, ρ(pentacene) is that of the non-interacting pentacene monolayer and ρ(Al) is that of the non-interacting Al substrate (all atoms are fixed at the same positions as in the combined system). Red colour indicates regions of higher electron density and blue ones of lower density. For B site adsorption, at an isovalue of Δρ = 0.005 *e*/Bohr^3^ we can clearly observe in Figure 2c accumulation of electron density between the two central carbon atoms (C1 and C1’) and the two Al atoms underneath them confirming the bonding of the molecule at that specific site. At a lower isovalue (0.002 *e*/Bohr^3^), as depicted in Figure 2a,b we can see that such charge originates from the electronic density depletion in the close proximity of the C–Al bond, with overall electron transfer from the surface to pentacene. An excess electronic charge of Δ*Q* = 0.56*e* is calculated on the adsorbed molecule, by means of Löwdin charge partitioning scheme [29–30]. One also observes (see the red regions in Figure 2a,b) electron accumulation between the atoms C3, C3’ and the surface, while other C atoms at larger distances are clearly less interacting with the surface. Intramolecular charge displacements in Figure 2a show electronic charge accumulation between the atoms C3–C2–C2’–C3’. It has to be stressed that at the same isovalue, no charge restructuring is visible for the flat molecule at the T site and hence a lower isovalue of 0.001 *e*/Bohr^3^ is chosen in Figure 2d to plot a much less localized charge displacement amounting to Δ*Q* = 0.20*e*.

**Figure 3:**
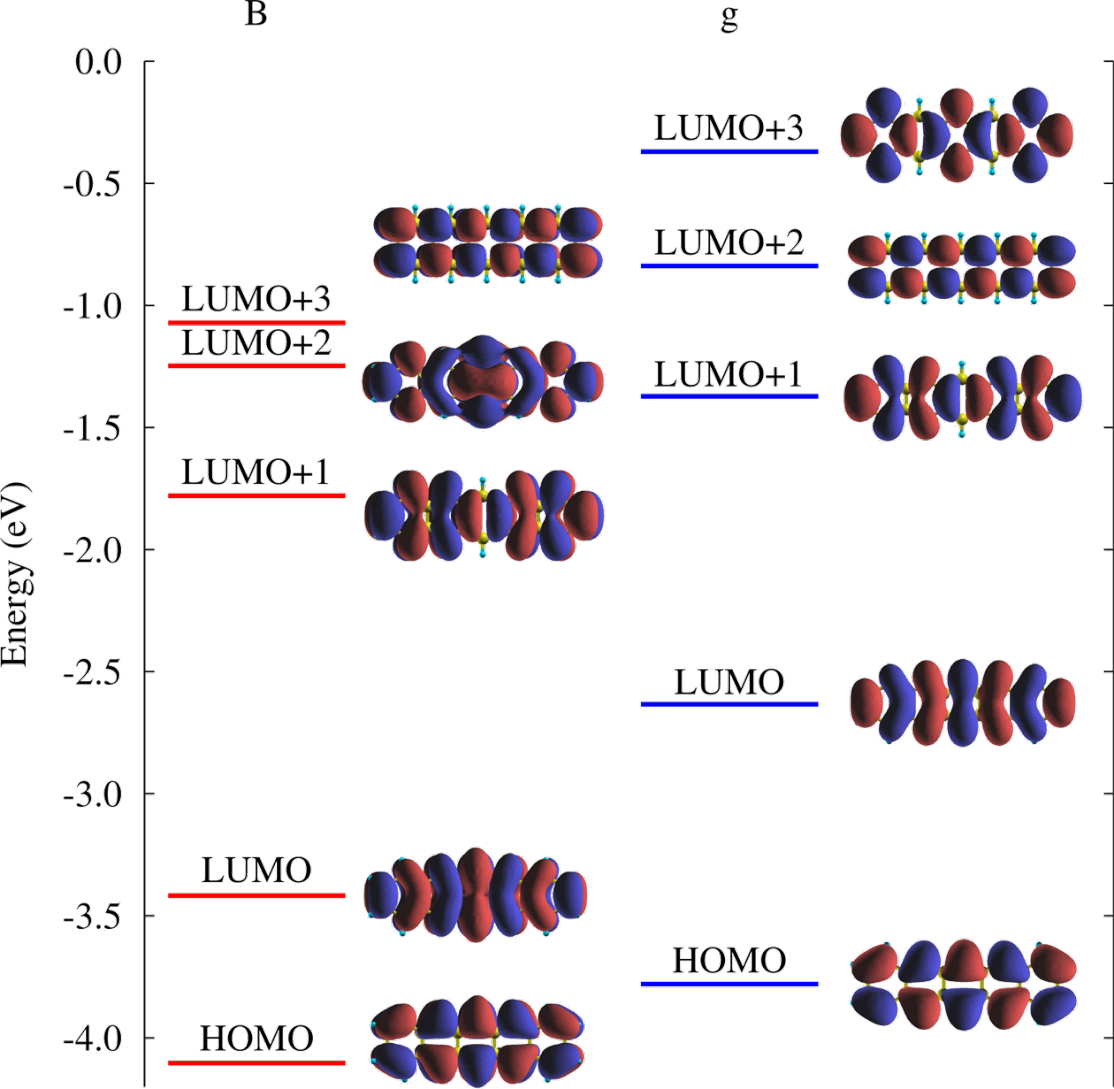
Comparison between the Kohn–Sham eigenvalues of the V-shaped gas phase pentacene with coordinates extracted from the bridge site adsorption (B) and the undistorted gas phase one (g). The corresponding orbital wave functions are also shown for each case.

The structural change in the V-shape of adsorbed pentacene induces alterations in the molecular orbitals, which are best perceived by looking first at the gas-phase molecule, but with the same geometry as that in the B site. In particular, the Kohn–Sham eigenvalues for bent and flat molecules are compared in Figure 3. One of the major features emerging is the reduction in the HOMO–LUMO gap of the free V-shaped molecule by 0.5 eV which decreases further for adsorption at the B site due to electron transfer. Upon adsorption, these states broaden and spread as a result of the substantial hybridization with the Al surface states. In particular for the LUMO an appreciable filling was observed as this state displays an energy range as large as 4 eV with its main peak below the Fermi energy level [15]. From the results in Figure 3 we add that the orbital corresponding to the LUMO+2 of the undistorted free molecule becomes the LUMO+3 of the V-shaped gas phase molecule (also compare with Table 2 below). This point is relevant in explaining the NEXAFS features in the following.

### XPS

**Figure 4:**
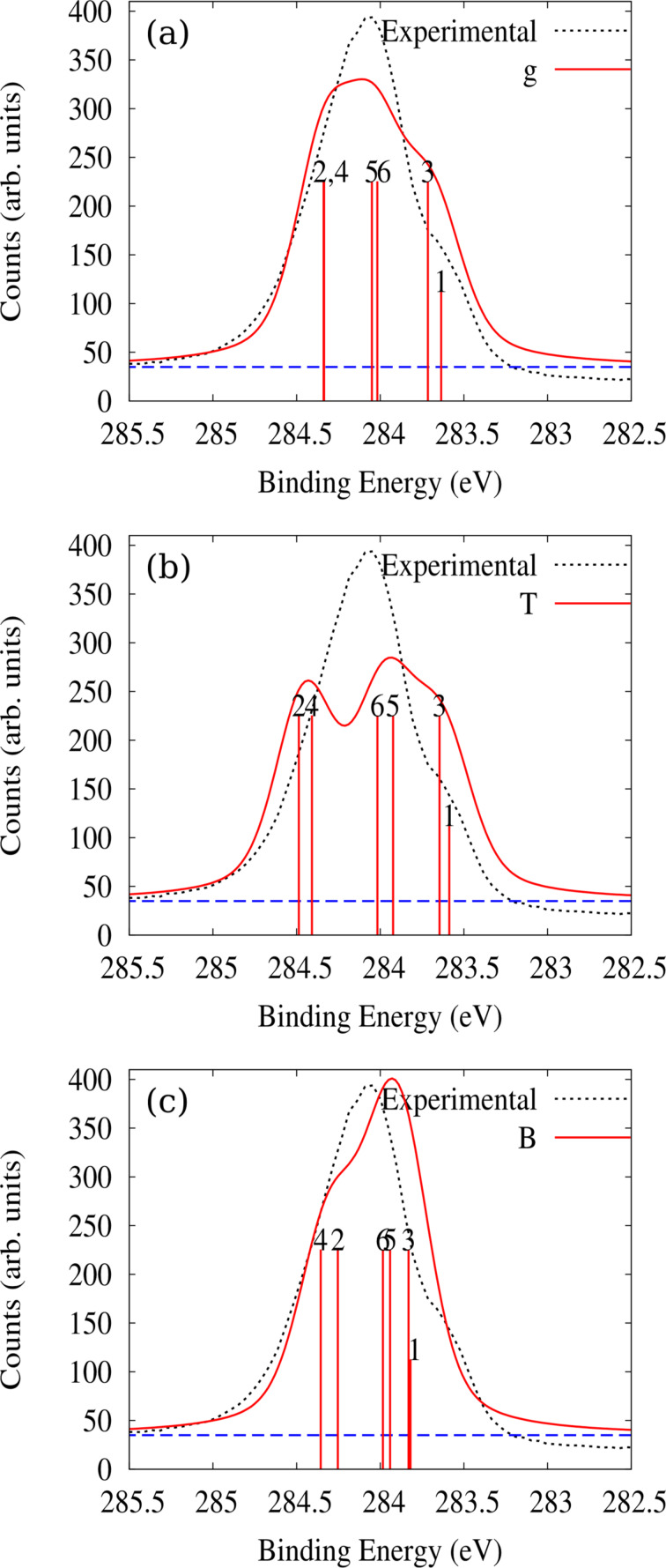
Experimental C 1s XPS spectrum for pentacene/Al(001) compared with the simulated spectra of (a) free pentacene, g, (b) pentacene at the T site and (c) pentacene at the B site. The individual contributions from the six non-equivalent carbon atoms are shown as vertical bars. These are convoluted with pseudo-Voigt profiles with 0.52 eV Lorentzian and 0.36 eV Gaussian full widths at half maximum and offset by a constant (see the dashed blue line) to obtain the simulated spectra (solid red line).

To understand the XPS features we connect the calculated core level shifts (CLS) to the screening charge of the system. The CLS are computed as the difference between the total energy of the system in the presence of a full core hole on the different non-equivalent C atoms and its weighted average taking into account their multiplicity. In Figure 4 we plot the core level C 1s photoemission spectra (XPS) obtained by experiment, and by simulations for free undistorted pentacene and pentacene adsorbed at T and B sites. Calculated initial state-binding energies are indicated as vertical bars with height proportional to the multiplicity of the non-equivalent carbon atoms (see Figure 1). These vertical bars when broadened (here with pseudo-Voigt profiles having 0.52 eV Lorentzian and 0.36 eV Gaussian full width at half maximum) determine the simulated XPS spectra reported in Figure 4. We remark that the use of a pseudopotential scheme does not allow us to access the absolute energy values and hence the simulated spectra are aligned to the experimental ones. The C 1s CLS spectrum computed in the gas phase [31], Figure 4a, is already able to capture the main features of the experimental result for adsorbed pentacene [15]. When we consider pentacene adsorbed on Al(001) at the T site, Figure 4b, the agreement actually worsens as the spectrum becomes too broad. Conversely, a good agreement is eventually observed for such V-shaped molecule on the B site, see Figure 4c, where the CLSs are smaller, further supporting the structural model.

The differences in the spectra reported in Figure 4 can be understood stepwise as follows. When the molecule is adsorbed almost undistorted at the T site, it may be affected by the surface coupling through several effects [32]. First, the full molecule is subjected to an effective potential due to the Al surface that influences similarly all atoms in the planar configuration and results in no net CLS. Second, the surface electrons screen the perturbation of the core hole, which is at the same distance in all cases determining, a net electron transfer, which may induce changes in the CLS. This effect can be estimated by performing additional simulations for the free undistorted molecule to calculate the shifts in binding energies as a function of a given excess electronic charge uniformly distributed. Such an excess charge results in a more negative shift of the binding energy for atoms C1, C3 and C5 and a more positive one for the C2, C4 and C6 energies, providing a broader spectrum in the charged molecule case (not shown in this paper) than in the gas phase neutral one. In particular, the C1–C2 binding energy difference amounts to 0.70 eV in the gas phase for neutral molecules and increases to 0.76 eV (0.84 eV) with 0.2 (0.5) additional electrons, in qualitative agreement to the value of 0.86 eV computed at the T site. Indeed, comparing Figure 4a and Figure 4b we point out that the excess charge is unevenly redistributed in the molecule, i.e., more electron density around C1, C3 and C5 and less around C2, C4 and C6, see also the LUMO amplitude in Figure 3. This reduces/increases the binding energy of 1s electrons in the first/second group of atoms, respectively [32].

**Figure 5:**
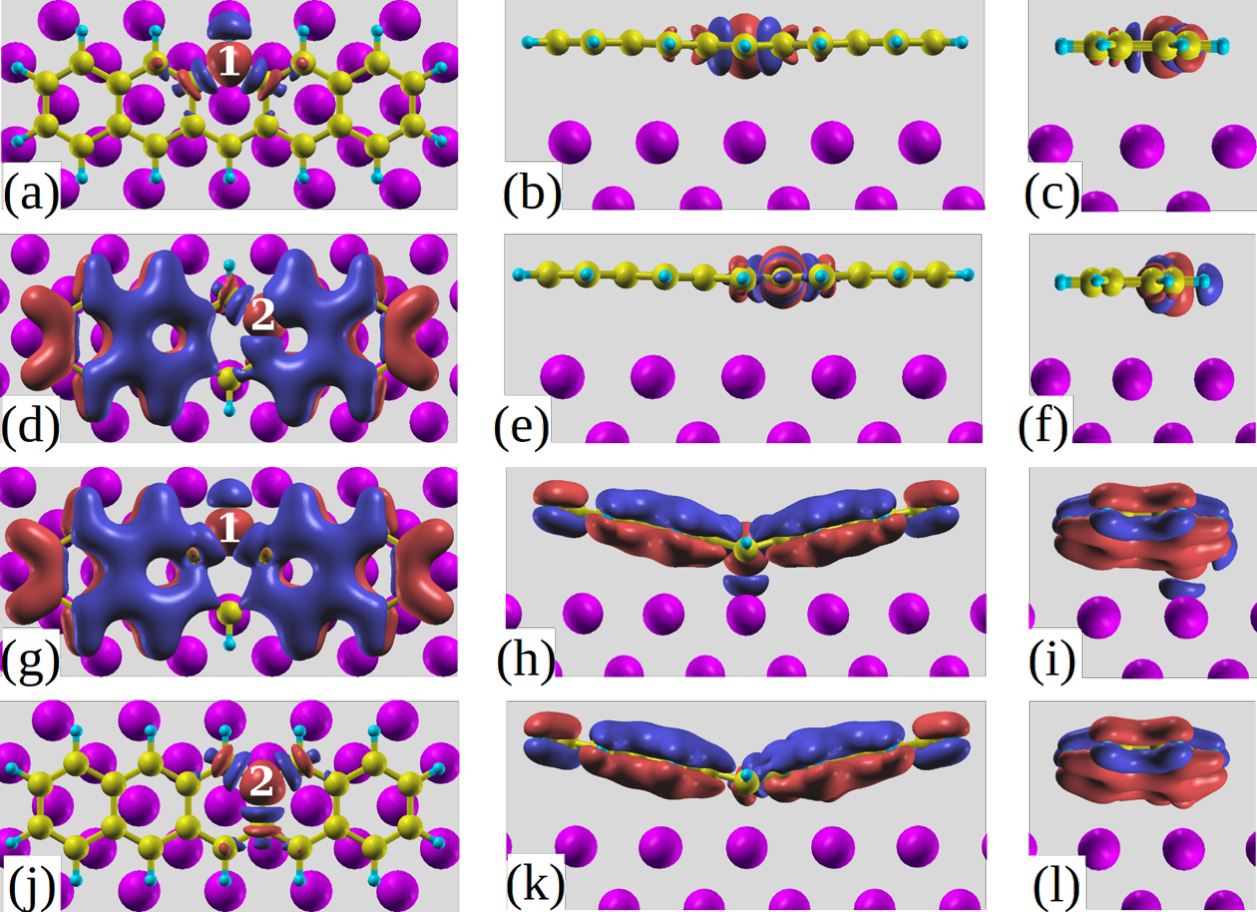
Top and side views of the screening charges in the presence of a full core hole on (a,b,c) C1 at the T site; (d,e,f) C2 at the T site; (g,h,i) C1 at the B site and (j,k,l) C2 at the B site for an isodensity value of 0.005 *e*/Bohr^3^.

This simple argument alone cannot explain the narrowing of the spectrum as we move from the T to the B site, where charge transfer upon adsorption is even higher but the C2–C1 binding energy difference amounts to 0.43 eV only (0.54 eV for C4–C1). Here the additional effect exists that the potential of the aluminum surface experienced by the various carbon atoms, is not a constant any more as the C atom heights differ by up to 1.35 Å. However, a non monotonic dependence on distance is observed, as a similar variation with respect to C2 is computed for C1 and C3 which are at smaller and larger heights respectively. Hence, the variations in screening offered by the system for the hole at the various sites must play an important role. To visualize such variations, we evaluated the screening charge following the C 1s level ionization, which we define as: Δρ^*^ = ρ(pentacene^fch^/Al) − ρ(pentacene/Al), where ρ(pentacene^fch^/Al) is the total charge of the combined system with a full core hole on the selected C atom, and ρ(pentacene/Al) is that of the combined system in its ground state. The same is compared in Figure 5 for the T and B sites, in the presence of a full core hole on atoms C1 and C2. We observe that the screening charge at the T site is similar for the two atoms C1 and C2 (Figure 5a–f), when seen from the excited atom, and that the largest charge displacements are localized in its proximity. Conversely, Δρ^*^ for the molecule at the B site (Figure 5g–l) extends throughout the molecule as it also involves large contributions from the π system polarizing towards the surface, because the planar symmetry of pentacene is lost at the B site. Note in particular the region of electron depletion (blue coloured) that is observed in Figure 5h,i just above the Al atom located below the 1s-excited C1: such depletion corresponds to a reduction of the C–Al bond shown in Figure 2c, and is absent in Figure 5k,l for excitation on C2. Hence, a larger destabilization of adsorption is expected for the core hole in C1 than in C2. Therefore the presence of core hole reduces the binding energy of C2 and increases that of C1 in agreement with a reduction in the difference between their core level binding energies thereby determining a narrower spectrum for B site adsorption as seen in Figure 4c and compensating the effects of electron transfer.

### NEXAFS

**Figure 6:**
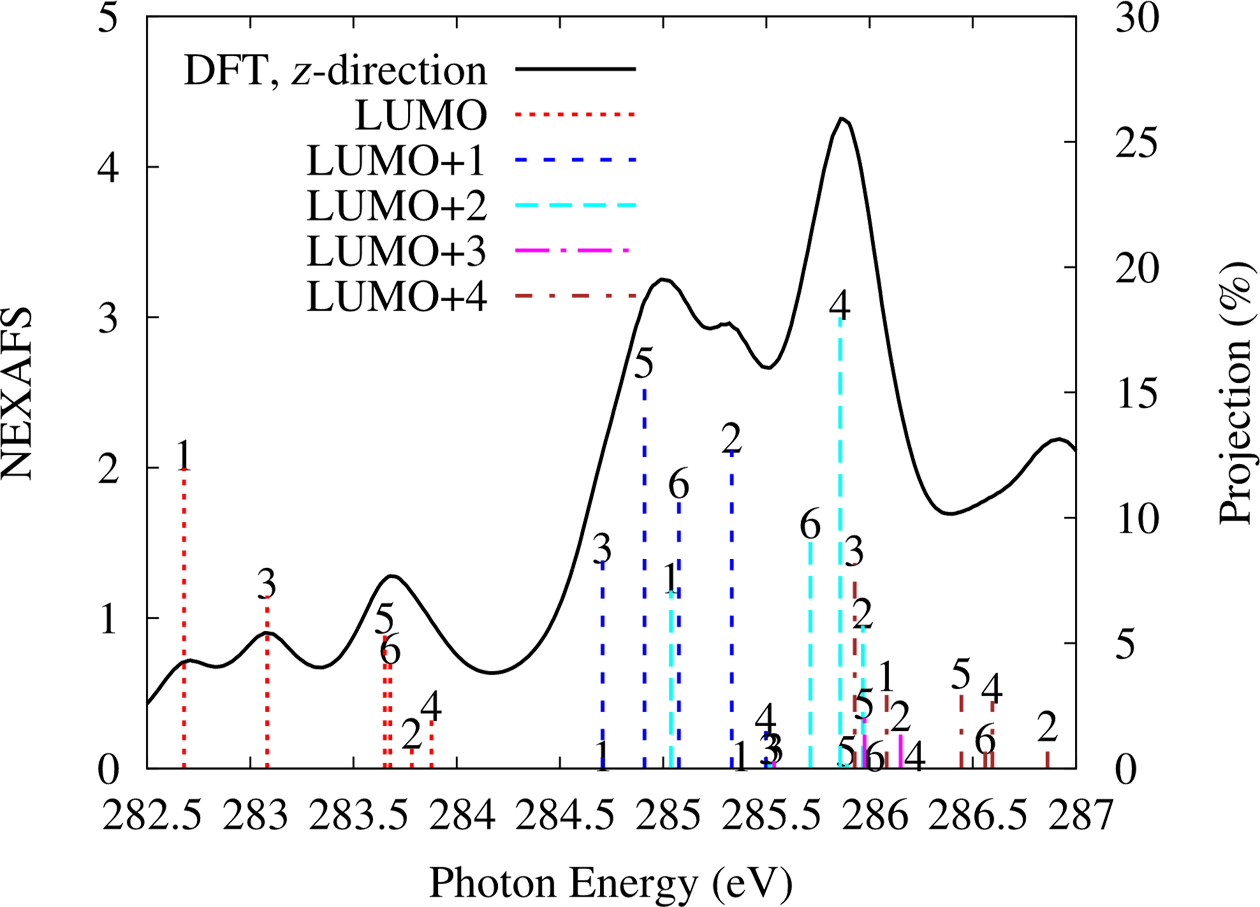
Simulated NEXAFS of the gas phase V-shaped pentacene from the B-site along the *z*-direction of the field, perpendicular to the molecular plane. For excitations from each C atom, the vertical bars numbered correspondingly show the projection of the various molecular orbitals, computed with a half core hole in that atom, on the 2p*_z_* state of the same carbon atom, and are referred to the axis on the right (in %).

We wish now to relate the simulated NEXAFS spectrum of pentacene/Al(001) with the electronic properties of the system. The calculation of NEXAFS is performed with half a core hole in the carbon 1s orbital. It is convenient to consider first a molecule in the gas phase but retaining the V-shaped geometry as in Figure 3. In particular, let us focus on the transitions to the lowest lying LUMO states with π^*^ symmetry, which characterize the low-energy part of the spectrum for the different C atoms. This for photon electric field along the *z*-direction, i.e., perpendicular to the plane of the molecule is shown in Figure 6 as a solid line. Owing to the lack of absolute energy reference from the calculation, this spectrum is arbitrarily offset so as to align it with the most prominent features in the spectra measured for absorbed molecules (to be presented subsequently). The contributions by individual excitations (initial and final states) is also marked in Figure 6 by vertical bars displaying the projected amplitude of the final state on the p*_z_* atomic states of the absorbing atoms, which produces a good qualitative description of the NEXAFS spectrum [31,33]. One can see two visibly separated broad features with multiple peaks. The first one (spanning the energy range between 282.5 eV and 284 eV) is completely due to the core–electron excitations to the LUMO of pentacene. The first peak of the second broad feature lying between 284.5 and 285.5 eV is mainly constituted by the LUMO+1 excitations and partly also by the LUMO+2 ones. The larger energy contributions to the second broad feature are from higher lying LUMO+*i* orbitals. One has to recall that the presence of a half core hole affects the molecular orbitals resulting into their intermixing and hybridization, as compared to the ground state. To show this, in Table 2 we report the overlap between the molecular orbitals of the excited V-shaped molecule in gas phase with those of the ground state of the free molecule (g), which are referred as former LUMOs, selecting as a representative example that of excited C1.

**Table 2:** Overlap of the molecular orbitals of the gas phase pentacene with a half core hole (hch) at C1 in the V-shaped geometry, with those of the free molecule in the ground state, g. Values below 0.5% are not reported here.

C1^hch^	orbitals of the free molecule in the ground state (g)			

	LUMO	LUMO+1	LUMO+2	LUMO+3

LUMO	77.2%	—	—	5.6%
LUMO+1	—	89.2%	—	—
LUMO+2	11.0%	—	—	51.4%
LUMO+3	—	—	86.5%	—
LUMO+4	—	—	—	9.5%
LUMO+5	—	—	—	16.8%

We can observe that the presence of a half core hole has not affected the LUMO but has significantly altered the higher lying LUMOs as also found for perylene derivatives [33] (here, from the LUMO+2). It is noteworthy that a free pentacene molecule has a nodal plane for (former) LUMO+1 and LUMO+2 along the central carbon atoms C1 and C1’ (see Figure 3) so no contribution to NEXAFS would be expected by transitions to these molecular orbitals from the 1s orbital of C1 at variance with the result in Figure 6 of a non-negligible contribution from the transition from the C1 1s to the LUMO+2. This is because we verified that the LUMO+2 and LUMO+3 orbitals get interchanged due to the V-shaped bending of pentacene already in the ground state, where its structural deformation causes the former LUMO+2 to shift to a higher energy than that of LUMO+3, which is retained also with the core hole. Indeed, now the LUMO+2 displays contributions by the former LUMO and LUMO+3 (see Table 2). Furthermore, we can see that LUMO+3 in Figure 6 has no weight on C1, as it originates mostly from the LUMO+2 of the undistorted free molecule which has moved to higher energy. In the case of adsorbed pentacene, the molecular orbitals broaden due to hybridization with the substrate states extending into overlapping energy ranges, hampering a similar analysis in terms of final states.

**Figure 7:**
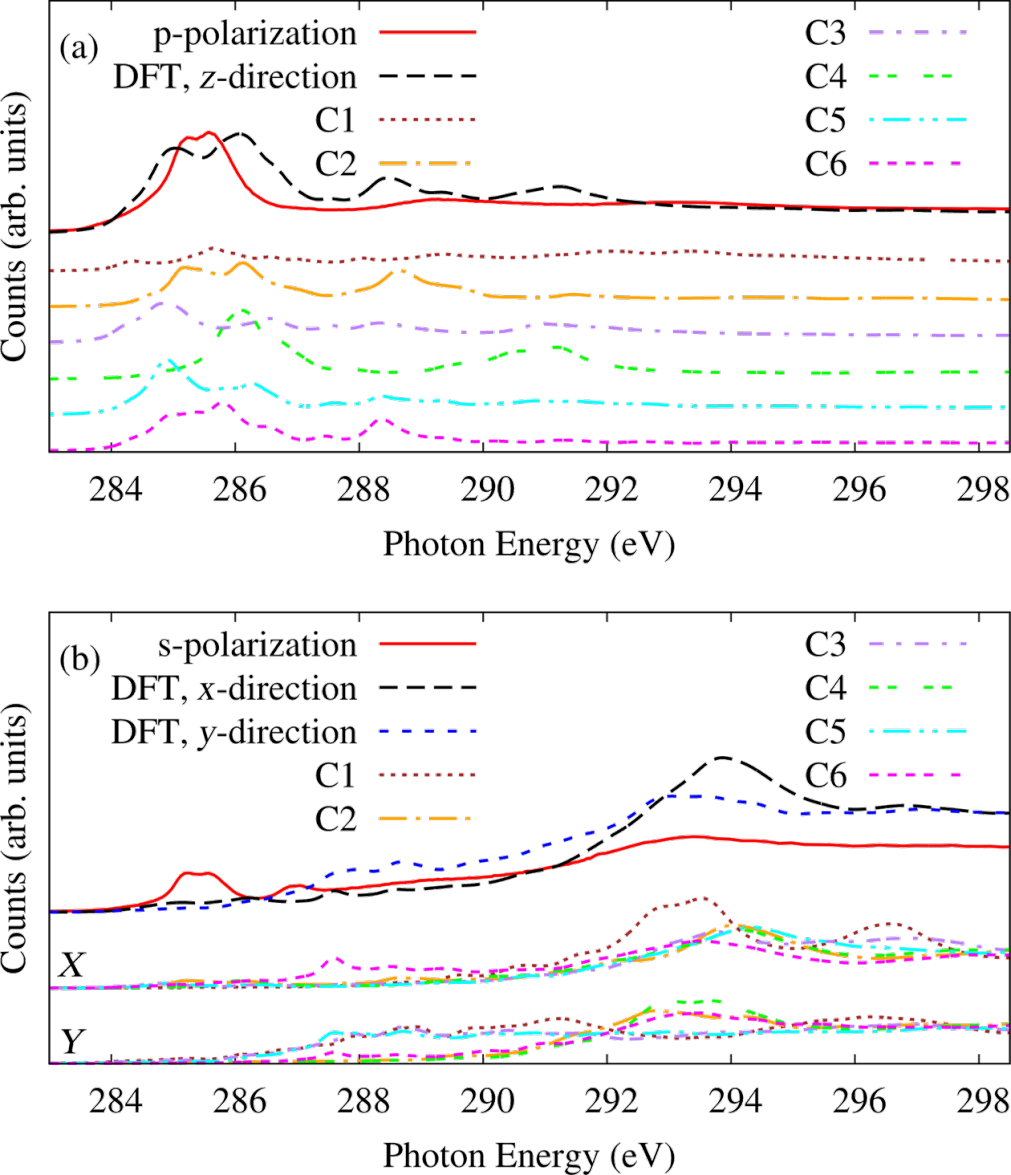
(a) Analysis of the initial state effects in the simulated NEXAFS spectrum and its comparison with the experimental one measured in p-polarization (electric field along *z*, i.e., perpendicular to the surface). (b) Same as (a), in s-polarization. Simulations are shown for electric field in the *x*- and *y*-directions, i.e., along the long and short molecular axes.

Figure 7 shows the simulated NEXAFS spectra for pentacene adsorbed at the B site in (a) p-polarization with the electric field perpendicular and (b) s-polarization with the electric field parallel to the surface. The long molecular axis is directed along the *x*-direction and the short one along the *y*-direction. There, we show the spectrum decomposed in terms of initial state effects, and we compare it to the experimental result (dashed line) [15]. Looking at the upper panel (a) of Figure 7, we can see a nice agreement between the experimental and simulated p-polarization (solid line) spectra. The simulated spectrum is arbitrarily aligned to the most prominent double peaked experimental feature centered at 285.5 eV which can be attributed to the π^*^ resonances. The first peak of the π^*^ resonance consists of contributions from all the atoms except C1 and C4. The second peak has contributions from all the atoms but with a larger weight from C4. It can be observed that the π^*^ resonances in Figure 7 have narrowed down compared to those of the V-shaped gas phase pentacene in Figure 6. It was mentioned before that the V-shaped structural deformation alone reduces the HOMO–LUMO gap of the free molecule by 0.5 eV which facilitates the filling of LUMO by getting electronic charge from aluminum and is hence absent in the NEXAFS spectrum [15]. In pentacene, C1 has the highest weight on LUMO but instead a node for LUMO+1 as can be seen in Figure 3. Hence, the absence of a significant contribution from C1 in the first peak of the π^*^ resonances in Figure 7 further confirms that it corresponds to the transitions to the LUMO+1 rather than the LUMO of the molecule.

Now let us look at the σ^*^ resonances of Figure 7b where the simulated NEXAFS computed for the molecule at the B site along the *x*- and *y*-directions (long and short molecular axes) are compared to the experimental NEXAFS in s-polarization. The experimental s-polarization has been averaged over the [110] and [001] surface azimuths, which provide very similar results as differently oriented domains are sampled. The experimental spectrum still displays some π^*^ resonance leftover centered at around 285.5 eV. This effect is not completely reproduced in the simulated spectrum even if we observe some non-zero contributions, which are due to the V-shaped molecules forming an angle of about 13° with the surface plane. Looking at the main σ^*^ peak centered at around 294 eV, we observe a small energy splitting between transitions with the electric field along the long and short molecular axes. This phenomenon called the azimuthal dichroism is much reduced, as compared to that of the free molecule. In the latter case, such energy splitting amounts to about 2 eV and was attributed equally to the intrinsic asymmetry of the molecule and to the shorter C–C bond lengths along the long molecular axis than that along the short one [31]. For adsorbed V-shaped molecules, the bond lengths along the two directions are more similar, as we reported in Table 1. Still the molecule being inherently anisotropic determines the residual azimuthal dichroism reduced to approx. 1 eV. Reporting the initial state contributions in this energy range, the *x*-direction spectrum has largest contributions by the core excitations from C1 whereas the *y*-direction one mainly by those from the carbon atoms C2, C4 and C6.

## Conclusion

The pentacene/Al(001) system was studied by means of DFT methods in order to understand how the electronic structure of the V-shaped adsorbed molecule affects the XPS and the NEXAFS results. The rationale of the most stable configuration, the bridge one, where the molecule adsorbs by bending into V-shape with its long molecular axis along the [110] direction on the Al(001) with a V-angle of 155°, has been accounted for. By analyzing the molecule–surface bond charge and how this is modified to screen the core level excitation, we demonstrate that the similarity of the observed XPS spectrum for the adsorbed molecules results from the compensation between a line shape broadening induced by charge transfer, and a narrowing due to excitation site dependent screening. The latter effect would be absent for non-physical undistorted molecules. NEXAFS spectra, resolved into individual atomic initial states, show no contribution by the LUMO and provide evidence for hybridization and interchange of molecular orbitals facilitated by the V-shape. A smaller azimuthal dichroism in NEXAFS associated with the energy splitting of the sigma resonances, is computed and explained in terms of modified C–C bond lengths.

## Computational Methods

The DFT calculations of the pentacene/Al(001) system were carried out using the Quantum-ESPRESSO package [34]. We choose the GGA as proposed by Perdew, Burke, and Ernzerhof (PBE) [35] for the exchange correlation functional. Plane waves and ultrasoft pseudopotentials generated with the Rappe, Rabe, Kaxiras, and Joannopoulos scheme [36] with a planewave cutoff of 27 Ry are used for relaxation and total energy calculations. The aluminum (001) surface is modeled by a slab with three layers of atoms, in a rectangular (8 × 5) surface unit cell. The repeated slab method is used with a vacuum space of 20 Å in the *z*-direction separating adjacent slabs. The surface Brillouin zone is integrated using a Monkhorst–Pack [37] set of special 2 × 3 grid of k-points and Methfessel–Paxton smearing [38] with a broadening of 0.02 Ry. The van der Waals interactions between the molecule and the surface are taken into account by adding semi empirical London dispersion forces in the Grimme approach [39] excluding the interactions within the Al atoms. The coordinates of pentacene deposited on only one side of the slab as well as the first layer of Al atoms were optimized as presented in Baby et al. [15] and are shown in Figure 1 for selected configurations (B and T sites). Modeling of the adsorption behavior of pentacene on the reconstructed Al surface has not been carried out as the molecule-induced reconstruction was found to be incommensurate and also because the accurate information about the surface structure is not available from the experiments. However calculations for the reconstructed surface are not expected to affect our computed XPS and NEXAFS spectra which are in very good agreement with experiments [15].

The XPS spectrum is computed in terms of the core level shifts (CLS) as defined in the text [40–41]. We remark that, differently from free molecules, the core hole in adsorbed molecules is eventually neutralized by the valence electrons from the metal substrate, so a globally neutral cell was used [42]. The corresponding core level shifts are computed with reference to the Fermi level of the substrate, as obtained in the experiments. The NEXAFS spectra are simulated using the xspectra code in Quantum-ESPRESSO [43–44]. We considered a half core hole in the carbon 1s orbital following the transition-potential approach introduced by Triguero et al. [45] as such calculations can reproduce the main features of the spectral profiles [31,33]. For XPS and NEXAFS calculations, the core level excitations were modeled by carbon pseudopotentials with a full and half core hole, respectively, in the 1s orbital (requiring a higher plane wave cutoff of 59 Ry). By the pseudopotential approach, absolute transition energies are not accessible and as a consequence the simulated spectra have been aligned to the experimental ones.
